# Expression of 14-3-3 protein isoforms in mouse oocytes, eggs and ovarian follicular development

**DOI:** 10.1186/1756-0500-5-57

**Published:** 2012-01-23

**Authors:** Santanu De, Jennifer L Marcinkiewicz, Srinivasan Vijayaraghavan, Douglas Kline

**Affiliations:** 1 Department of Biological Sciences, Kent State University, Kent, OH 44242, USA

## Abstract

**Background:**

The 14-3-3 (YWHA) proteins are a highly conserved, ubiquitously expressed family of proteins. Seven mammalian isoforms of 14-3-3 are known (β, γ, ε, ζ, η, τ and, σ). These proteins associate with many intracellular proteins involved in a variety of cellular processes including regulation of the cell cycle, metabolism and protein trafficking. We are particularly interested in the role of 14-3-3 in meiosis in mammalian eggs and the role 14-3-3 proteins may play in ovarian function. Therefore, we examined the expression of 14-3-3 proteins in mouse oocyte and egg extracts by Western blotting after polyacrylamide gel electrophoresis, viewed fixed cells by indirect immunofluorescence, and examined mouse ovarian cells by immunohistochemical staining to study the expression of the different 14-3-3 isoforms.

**Results:**

We have determined that all of the mammalian 14-3-3 isoforms are expressed in mouse eggs and ovarian follicular cells including oocytes. Immunofluorescence confocal microscopy of isolated oocytes and eggs confirmed the presence of all of the isoforms with characteristic differences in some of their intracellular localizations. For example, some isoforms (β, ε, γ, and ζ) are expressed more prominently in peripheral cytoplasm compared to the germinal vesicles in oocytes, but are uniformly dispersed within eggs. On the other hand, 14-3-3η is diffusely dispersed in the oocyte, but attains a uniform punctate distribution in the egg with marked accumulation in the region of the meiotic spindle apparatus. Immunohistochemical staining detected all isoforms within ovarian follicles, with some similarities as well as notable differences in relative amounts, localizations and patterns of expression in multiple cell types at various stages of follicular development.

**Conclusions:**

We found that mouse oocytes, eggs and follicular cells within the ovary express all seven isoforms of the 14-3-3 protein. Examination of the differential expression of these 14-3-3 isoforms in female germ cells and ovarian follicles provides the foundation for further investigating 14-3-3 isoform-specific interactions with key proteins involved in ovarian development, meiosis and oocyte maturation. This will lead to a better understanding of the individual functional roles of the 14-3-3 protein isoforms in mammalian oogenesis and female reproductive development.

## Background

Members of the 14-3-3 family are key proteins in a number of intracellular events, particularly those involving phosphorylation-dependent switching. The proteins bind to a diverse set of target proteins and alter cellular function by binding to and causing conformational changes in target proteins or modifying target protein interactions with other proteins. Of particular interest, 14-3-3 appears to be central to several aspects of vertebrate development and cell cycle regulation, including meiosis in amphibians [1,2]; however, the functions of 14-3-3 in mammalian reproductive organs and in gametes have not been completely elucidated. There is also interest in understanding the role of 14-3-3 proteins in the regulation of oogenesis and the cell cycle during oocyte maturation and in early development. In addition, 14-3-3 proteins, by their participation in the regulation of the cell cycle, apoptosis, and tumor suppression, are important in normal growth and development as well as in cancer [3].

The 14-3-3 proteins are a family of highly conserved, homologous proteins encoded by separate genes. The name for the protein family is tyrosine 3-monooxygenase/tryptophan 5-monooxygenase activation protein family (YWHA). The 14-3-3 name is still commonly used. There are seven mammalian isoforms of 14-3-3 encoded by seven different genes: β (*Ywhab*), γ (*Ywhag*), ε (*Ywhae*), ζ, (*Ywhaz*), η (*Ywhah*), τ (*Ywhaq*) and σ (*Sfn*) [4]. The 14-3-3 proteins exist as homo- or hetero-dimers [5,6]. It is known that different 14-3-3 isoforms can interact with the same ligand and so are somewhat interchangeable. Although different isoforms of 14-3-3 may bind the same protein, there are some indications that homodimers of different types or even heterodimers of 14-3-3 may have different roles in the regulation or sequestering of proteins [7-9].

The roles of 14-3-3 proteins in the ovary may parallel function in other tissues. For example, 14-3-3σ is expressed at lower levels in cancerous cells in a number of tissues including adenocarcinomas of the ovary [3,10]. However, specific descriptions of the roles of 14-3-3 proteins in the ovary are few. In female mammals, meiosis is initiated prenatally and oocytes remain arrested in an immature state at late prophase of the first meiotic division for long periods of time. This arrest is released as a result of the pre-ovulatory surge in luteinizing hormone and oocytes enter the first meiotic division cycle and arrest at metaphase II of meiosis to form the mature egg. It has been suggested that, in mammalian oocytes, 14-3-3 binds to and regulates the cell cycle control protein CDC25B phosphatase (cell division cycle 25 homolog B), as it does in amphibian oocytes, to hold the cell in prophase arrest [11]. In another case, we have shown that 14-3-3 interacts with phosphorylated PADI6, a key maternal effect protein, in mature eggs, but not with unphosphorylated PADI6 in immature oocytes [12]. While such interactions have been examined in part, more information about specific isoforms is needed. It is also clear that many more cellular processes in the ovary and in the female gametes might be regulated by 14-3-3. As a pre-requisite for understanding the role(s) of 14-3-3 in mammalian female reproductive development and oocyte maturation, we must understand which cell types express which individual isoforms. The present study explores the various isoforms of this protein and the characteristic patterns of expression in immature oocytes, mature eggs and in the various developmental stages of ovarian follicles in the adult mouse.

## Results and discussion

### 14-3-3 proteins in oocytes and eggs

Immature, prophase I-arrested mouse oocytes and mature, metaphase II-arrested eggs appear to express all seven 14-3-3 isoforms. Three approaches were used to determine if these cells contained each of the isoforms of 14-3-3. We examined the proteins in oocyte and egg extracts by Western blotting after polyacrylamide gel electrophoresis, viewed fixed cells by indirect immunofluorescence, and examined oocytes also by immunohistochemical staining of cells within ovarian sections. All three approaches relied on a panel of antibodies that has been shown to be specific for the various 14-3-3 isoforms. Martin and his colleagues [13,14] described the generation of the panel of antibodies and used them to detect the major brain isoforms of 14-3-3. They confirmed, by several methods, the high specificity of each of these antibodies, which is due to the fact that the epitope for each antibody is mainly in the N-acetylated amino terminus of the different peptide immunogens. The panel of 14-3-3 isoform-specific antibodies was also used to identify the isoforms of 14-3-3 proteins expressed in human dermal and epidermal layers [15] and in adrenal chromaffin cells [16].

### Presence and relative abundance of 14-3-3 isoforms in oocytes and eggs determined by Western blotting

**Figure 1:**
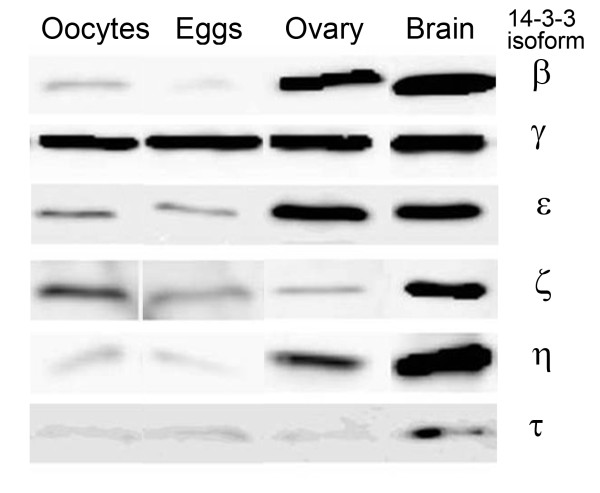
**Immunoblots identifying 14-3-3 isoforms in extracts from mouse oocytes, eggs and ovaries**. Proteins from cell lysates were separated by electrophoresis under reducing conditions, transferred to membranes and probed with antibodies directed against the 14-3-3 isoforms indicated. Each isoform shown was detected in lysates of 200 oocytes or 200 eggs. Protein extracts of ovaries and brain from adult mice are included for comparison. The 14-3-3 protein is approximately 30 kDa.

Western blots of extracts from 200 oocytes or 200 eggs indicate the presence of six of the seven isoforms (Figure 1). These six isoforms of 14-3-3 were also detected in ovarian protein extracts by Western blotting. We could not detect 14-3-3σ by Western blotting; however, it was identified in oocytes and eggs by immunocytochemistry and in ovarian follicle cells, including oocytes, by immunohistochemical staining. The inability to detect 14-3-3σ in Western blots may be due to the unsuitability of this antibody in recognizing a denatured antigen in our Western blotting procedure.

**Figure 2:**
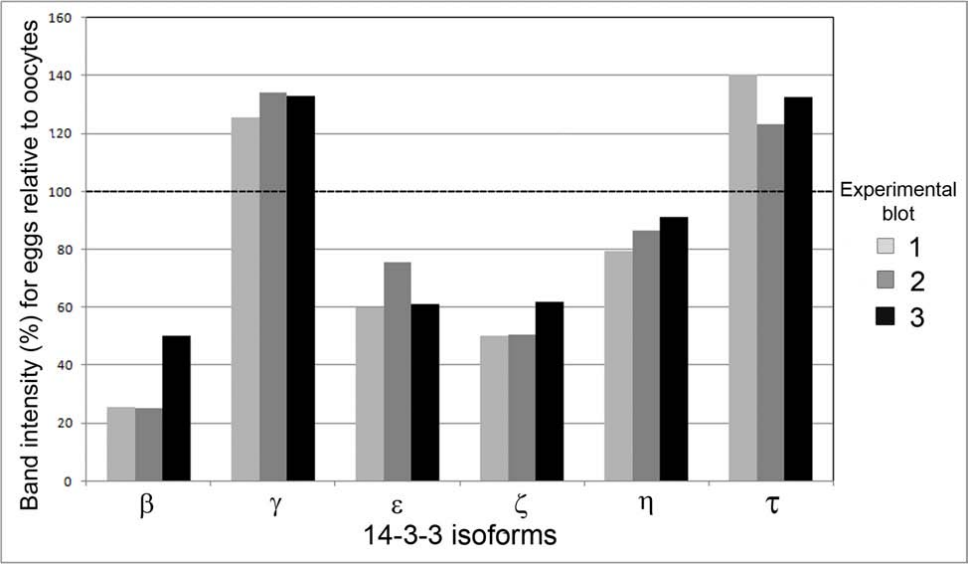
**Comparison of the relative abundance of individual 14-3-3 isoforms in immature oocytes and mature eggs**. The relative abundance of each individual isoform is based on the integrated densities of bands obtained from immunoblots (see Methods). Three experimental immunoblots were prepared for each of the six isoforms shown. In each experiment, for one of the isoforms, protein extracts from 200 oocytes and 200 eggs was analyzed after electrophoresis and immunoblotting on the same blot. For every blot, the density of the band for oocyte extracts was normalized to 100% and the density of the band for eggs is given as a percent with regards to the normalized oocyte value.

We were interested to determine the relative amounts of the 14-3-3 isoforms in immature oocytes and in mature eggs. Quantitative changes in total amount of specific 14-3-3 isoforms could provide insights into the regulation of oocyte maturation or other aspects of development. Such a comparison is possible as each experiment examines proteins from the same number of oocytes or eggs (200 cells) loaded onto two lanes of gel and the proteins are simultaneously transferred to a membrane and probed with the same antibody and Western blotting reagents. The qualitative comparison of one such experiment is shown in Figure 1. We repeated this experiment two additional times and made a relative comparison for each isoform (Figure 2). The proteins 14-3-3β, 14-3-3ε, 14-3-3η, and 14-3-3ζ appear in lesser amounts in mature eggs than in immature oocytes. For example, a marked decrease in the 14-3-3β isoform after maturation of oocytes into eggs is observed. On the contrary, amounts of 14-3-3γ and 14-3-3τ were found to increase following oocyte maturation. It should be noted that, in these experiments, it is only possible to make quantitative comparisons for a single isoform and not between different isoforms as the isoform-specific antibodies may have different affinities and therefore different intensities on a Western blot.

### Expression of 14-3-3 isoforms in oocytes and eggs determined by immunofluorescence

**Figure 3:**
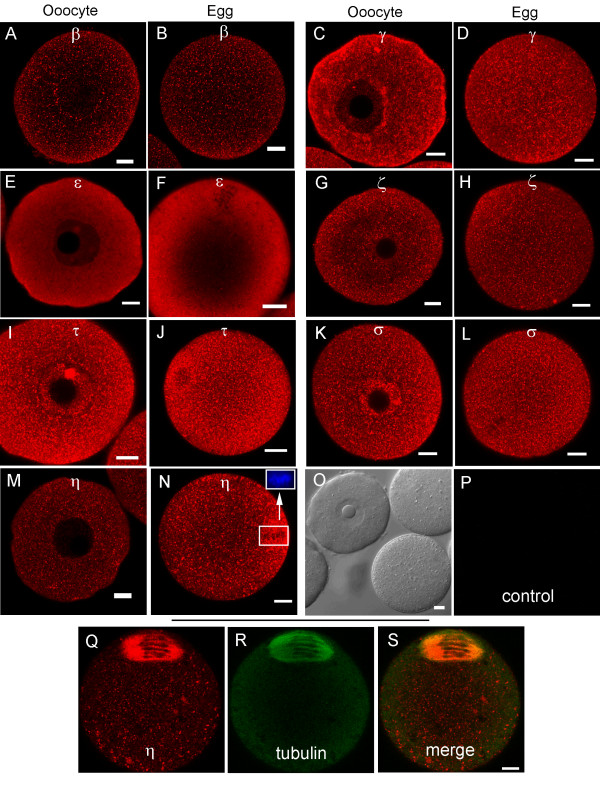
**Representative immunofluorescence images of 14-3-3 isoforms in oocytes and eggs isolated from adult mice**. (A, B) 14-3-3β. (C, D) 14-3-3γ. (E, F) 14-3-3ε. (G, H) 14-3-3ζ. (I, J) 14-3-3 τ. (K, L) 14-3-3σ. (M, N) 14-3-3η. Confocal sections with regions of red fluorescence indicating the corresponding isoforms studied (see Methods). The inset in N shows the same egg labeled blue with Hoechst DNA stain (non-confocal image) and confirms that the darker areas in this region of the larger image are condensed metaphase II chromosomes. Control cells were included for each isoform experiment and were imaged using the same confocal settings. Representative control oocytes and eggs are shown in bright-field (O) and fluorescence (P). 14-3-3η accumulates, in part, in the meiotic spindle in eggs as shown by simultaneous labeling with 14-3-3η (Q) and tubulin (R) antibodies. These sequential scans are merged (14-3-3η + tubulin) in (S). The scale bars represent 10 μm for all images.

Immunofluorescence microscopy confirms the presence of all seven 14-3-3 isoforms in oocytes and eggs (Figure 3). Oocytes and eggs were fixed in paraformaldehyde, permeabilized with detergent and incubated with isoform-specific antibodies for 14-3-3, followed by application of a fluorescently-labeled secondary antibody and viewed by scanning confocal microscopy. The subcellular distributions of the isoforms were found to vary from one isoform to another. For example, 14-3-3ε is expressed uniformly throughout the oocyte with some peripheral accumulation, and absent in the interior of the egg cytoplasm (Figure 3E-F). 14-3-3τ is distributed uniformly in oocytes and eggs, but is particularly absent along the inner nuclear membrane of all oocytes examined (Figure 3I-J). Isoforms 14-3-3β, 14-3-3γ and 14-3-3ζ exhibit a notable peripheral accumulation in oocytes, with a uniform distribution in eggs (Figure 3A-B,3C-D and 3G-H respectively). 14-3-3σ is found to be expressed in higher levels in nuclei of all oocytes studied, as compared to their cytoplasm, where it is uniformly dispersed with some accumulation selectively along one half of the cell (Figure 3K); however, the 14-3-3σ isoform shows a uniform distribution in eggs (Figure 3L). 14-3-3η is diffusely dispersed in oocyte with lesser distribution in the germinal vesicle than in the cytoplasm, but attains a uniform punctuate distribution with prominent accumulation in the region of the meiotic spindle in all eggs observed (Figure 3M-N).

Control oocytes and eggs incubated in secondary antibody alone displayed little background fluorescence at the same laser intensities and confocal imaging settings used for each immunofluorescence experiment (Figure 3O-P). No attempt was made to compare the relative fluorescence intensity of a particular isoform in oocytes or eggs with that of a different isoform, since the antibodies detecting the isoforms were all different.

These experiments are the first to examine all of the 14-3-3 proteins in mouse oocytes and eggs. Previous work suggested that multiple isoforms could be present, for example an examination of the maternal component of the zygotic polysomal mRNA population in mouse eggs and one-cell embryos revealed an increase in maternal mRNAs for 14-3-3β, 14-3-3γ, 14-3-3ζ, 14-3-3η and 14-3-3τ. The other isoform mRNAs were not examined in this paper [17].

It has been suggested that, in mammalian oocytes, 14-3-3 binds to and regulates the cell cycle control protein CDC25B phosphatase [11]. There is good evidence from studies of frog oocytes that 14-3-3 protein may act to hold the oocyte in prophase I arrest by binding to and localizing phosphorylated CDC25B in the cytoplasm [18]. Following the induction of oocyte maturation, CDC25B is thought to be dephosphorylated and released from 14-3-3, allowing it to participate in the activation of MPF which leads to germinal vesicle breakdown and the resumption of meiosis. It is not known which of the seven isoforms in the mammalian oocyte might be interacting with CDC25B; we have shown here that all are present in oocytes. The interaction of 14-3-3 with the cell cycle control protein CDC25B has been examined in mammalian somatic cells. There is strong evidence to indicate that 14-3-3β, 14-3-3ε, and 14-3-3σ bind to CDC25B and that 14-3-3β is responsible for sequestering CDC25B in the cytoplasm [11,19]. Future experiments will be needed to determine if the other 14-3-3 isoforms, which we have now found to be present as well in mouse oocytes, also interact with CDC25B and whether they are involved in the regulation of oocyte maturation.

Prominent localization of 14-3-3σ in the nuclei of oocytes (Figure 3I) is consistent with the observations that 14-3-3 proteins can shuttle through the nuclear membrane, but is in contrast with some observations in somatic cells in which it was noted that 14-3-3σ is more abundant in the cytoplasm than in the nucleus, while 14-3-3ζ is more abundant in the nucleus as compared to the cytoplasm [20].

Localization of 14-3-3η in the meiotic spindle (Figure 3N, Q, R and 3S) suggests a role for 14-3-3 in spindle assembly or cell cycle control. This is the first evidence for the localization of a specific 14-3-3 isoform in the metaphase II spindle of mouse eggs. It has been reported that 14-3-3ε and 14-3-3γ localize in the centrosome and mitotic spindle of some mouse somatic cells lines [21]. Additional functional studies are needed to determine if 14-3-3η plays a role in the formation or regulation of the meiotic spindle or chromosome separation in mammalian oocytes.

### Expression of 14-3-3 isoforms in ovarian cells determined by immunohistochemistry

As work on the role of 14-3-3 in ovarian development, oogenesis and cancer proceeds, it will be valuable to know which 14-3-3 isoforms are present and/or abundant in both the somatic cells and the germ cells within the ovary. We examined mouse ovarian follicular sections by immunohistochemical staining using isoform-specific antibodies and the Avidin: Biotinylated enzyme Complex (ABC) technique. The sections contained ovarian follicles at all stages of development. The ABC method relies on the high affinity of avidin for biotin and the method is known to produce minimal background staining in the absence of primary antibody [22,23]. Regions stained brown indicate presence of the 14-3-3 isoforms in contrast with regions counterstained blue. Again it is not possible to determine the relative amounts of distribution of a particular isoform in cells compared to other isoforms as the antibodies are different; nevertheless for a given isoform, variations in the intensities of staining indicate differences in relative amounts of expression among different cells in follicles and surrounding tissue.

**Figure 4:**
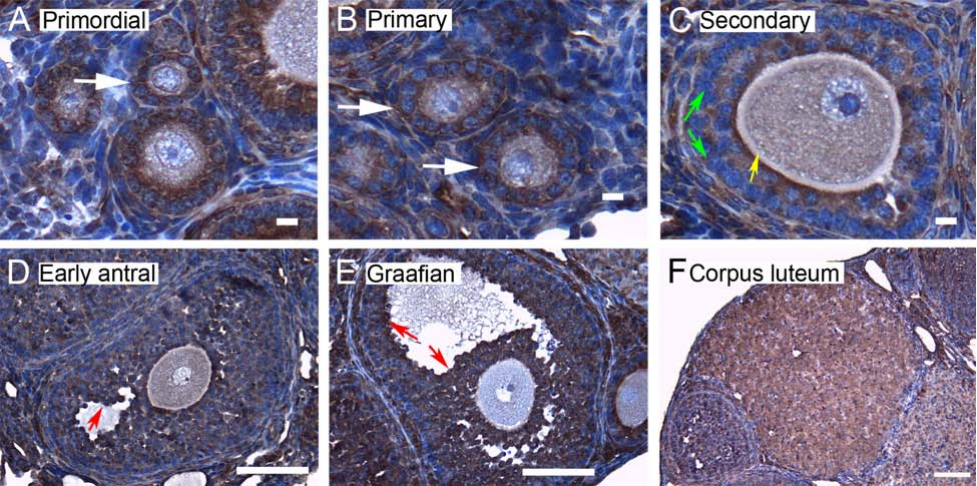
**Representative immunohistochemistry images of 14-3-3 β in the different stages of follicular development in ovarian sections**. Brown staining represents 14-3-3 β against regions counterstained blue with hematoxylin. (A) Primordial follicle. (B) Primary follicle. (C) Secondary follicle. (D) Early antral follicle. (E) Graafian (advanced antral) follicle. (F) *Corpus luteum*. White arrows indicate the primordial or primary follicles in (A and B). Note the weaker staining in mural granulosa cells in secondary follicles (C, green arrows), the more intense stain along the *zona pellucida *of the oocyte (C, yellow arrow), and the more intense staining in cells lining the antral cavity (D and E, red arrows). The scale bars represent 10 μm (A-C) or 100 μm (D-F).

**Figure 5:**
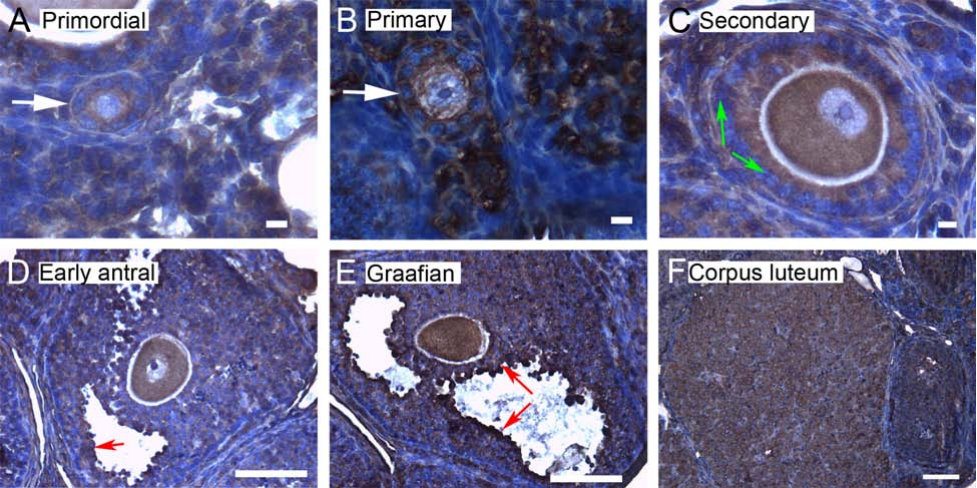
**Representative immunohistochemistry images of 14-3-3 γ in the different stages of follicular development in ovarian sections**. (A) Primordial follicle. (B) Primary follicle. (C) Secondary follicle. (D) Early antral follicle. (E) Graafian (advanced antral) follicle. (F) *Corpus luteum*. White arrows indicate the primordial or primary follicles in (A and B). Note the weaker staining in mural granulosa cells in secondary follicles (C, green arrows) and the more intense staining in cells lining the antral cavity (D and E, red arrows). The scale bars represent 10 μm (A-C) or 100 μm (D-F).

**Figure 6:**
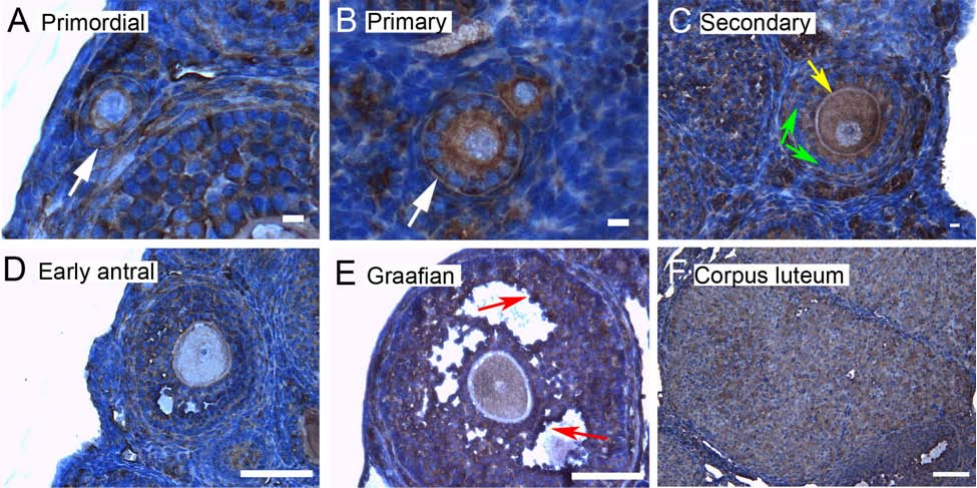
**Representative immunohistochemistry images of 14-3-3 ε in the different stages of follicular development in ovarian sections**. (A) Primordial follicle. (B) Primary follicle. (C) Secondary follicle. (D) Early antral follicle. (E) Graafian (advanced antral) follicle. (F) *Corpus luteum*. White arrows indicate the primordial or primary follicles in (A and B). Note the weaker staining in mural granulosa cells in secondary follicles (C, green arrows), the more intense stain along the *zona pellucida *of the oocyte (C, yellow arrow), and the more intense staining in cells lining the antral cavity (E, red arrows). The scale bars represent 10 μm (A-C) or 100 μm (D-F).

**Figure 7:**
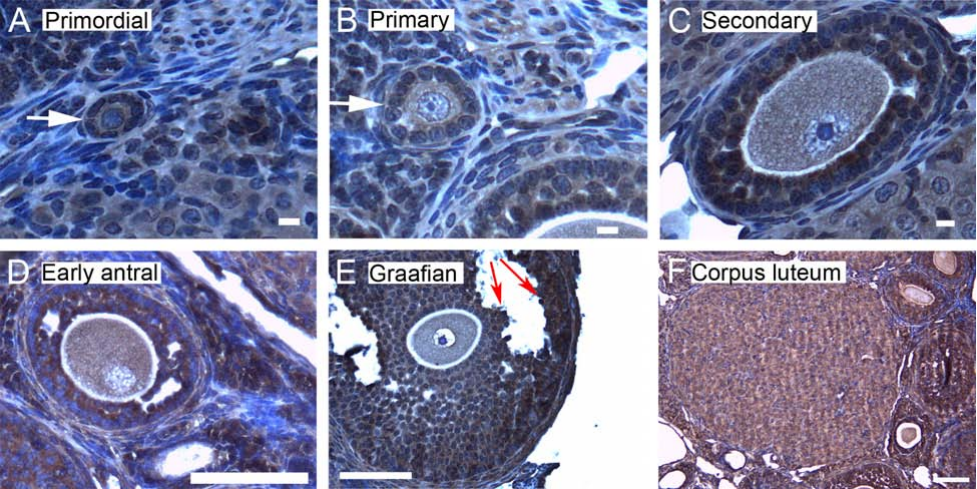
**Representative immunohistochemistry images of 14-3-3 ζ in the different stages of follicular development in ovarian sections**. (A) Primordial follicle. (B) Primary follicle. (C) Secondary follicle. (D) Early antral follicle. (E) Graafian (advanced antral) follicle. (F) *Corpus luteum*. White arrows indicate the primordial or primary follicles in (A and B). Note the more intense staining in cells lining the antral cavity (E, red arrows). The scale bars represent 10 μm (A-C) or 100 μm (D-F).

**Figure 8:**
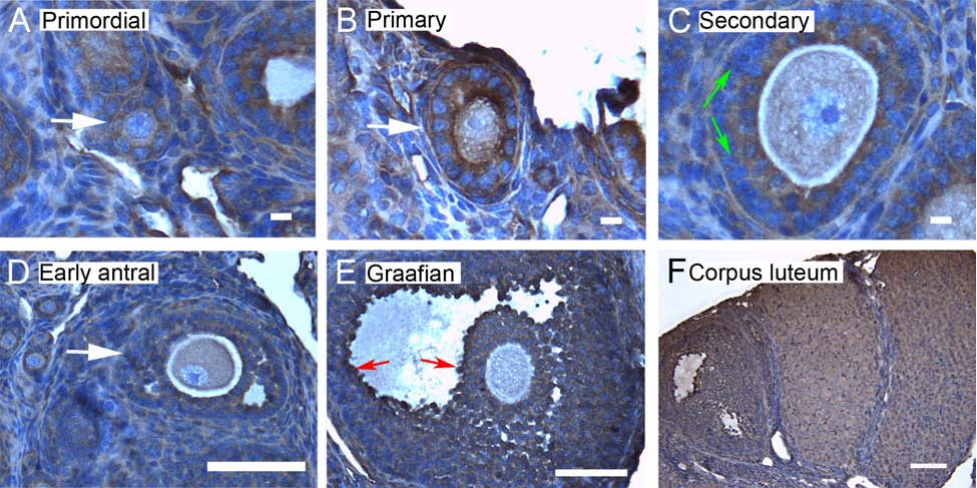
**Representative immunohistochemistry images of 14-3-3 η in the different stages of follicular development in ovarian sections**. (A) Primordial follicle. (B) Primary follicle. (C) Secondary follicle. (D) Early antral follicle. (E) Graafian (advanced antral) follicle. (F) *Corpus luteum*. White arrows indicate the primordial or primary follicles in (A and B). Note the weaker staining in mural granulosa cells in secondary follicles (C, green arrows) and the more intense staining in cells lining the antral cavity (E, red arrows). The scale bars represent 10 μm (A-C) or 100 μm (D-F).

**Figure 9:**
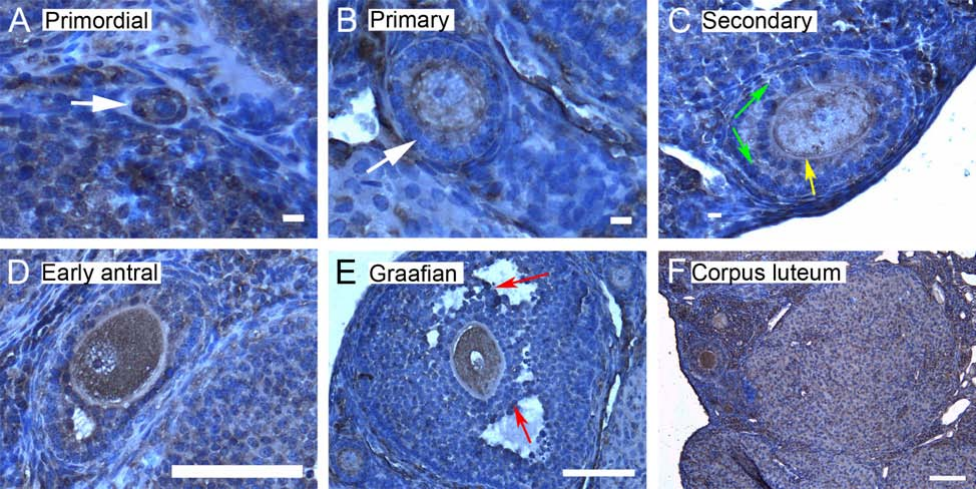
**Representative immunohistochemistry images of 14-3-3 τ in the different stages of follicular development in ovarian sections**. (A) Primordial follicle. (B) Primary follicle. (C) Secondary follicle. (D) Early antral follicle. (E) Graafian (advanced antral) follicle. (F) *Corpus luteum*. White arrows indicate the primordial or primary follicles in (A and B). Note the weaker staining in mural granulosa cells in secondary follicles (C, green arrows), the more intense stain along the *zona pellucida *of the oocyte (C, yellow arrow), and the more intense staining in cells lining the antral cavity (E, red arrows). The scale bars represent 10 μm (A-C) or 100 μm (D-F).

**Figure 10:**
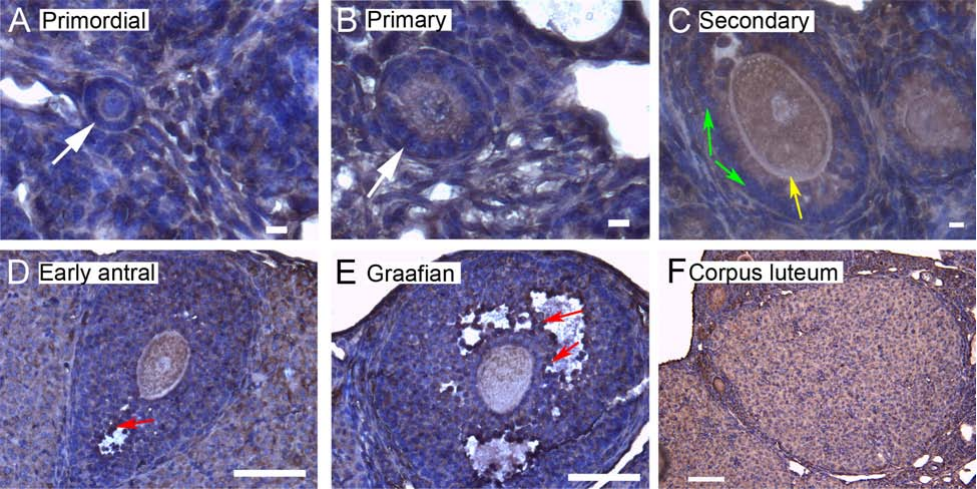
**Representative immunohistochemistry images of 14-3-3 σ in the different stages of follicular development in ovarian sections**. (A) Primordial follicle. (B) Primary follicle. (C) Secondary follicle. (D) Early antral follicle. (E) Graafian (advanced antral) follicle. (F) *Corpus luteum*. White arrows indicate the primordial or primary follicles in (A and B). Note the weaker staining in mural granulosa cells in secondary follicles (C, green arrows), the more intense stain along the *zona pellucida *of the oocyte (C, yellow arrow), and the more intense staining in cells lining the antral cavity (D and E, red arrows). The scale bars represent 10 μm (A-C) or 100 μm (D-F).

**Figure 11:**
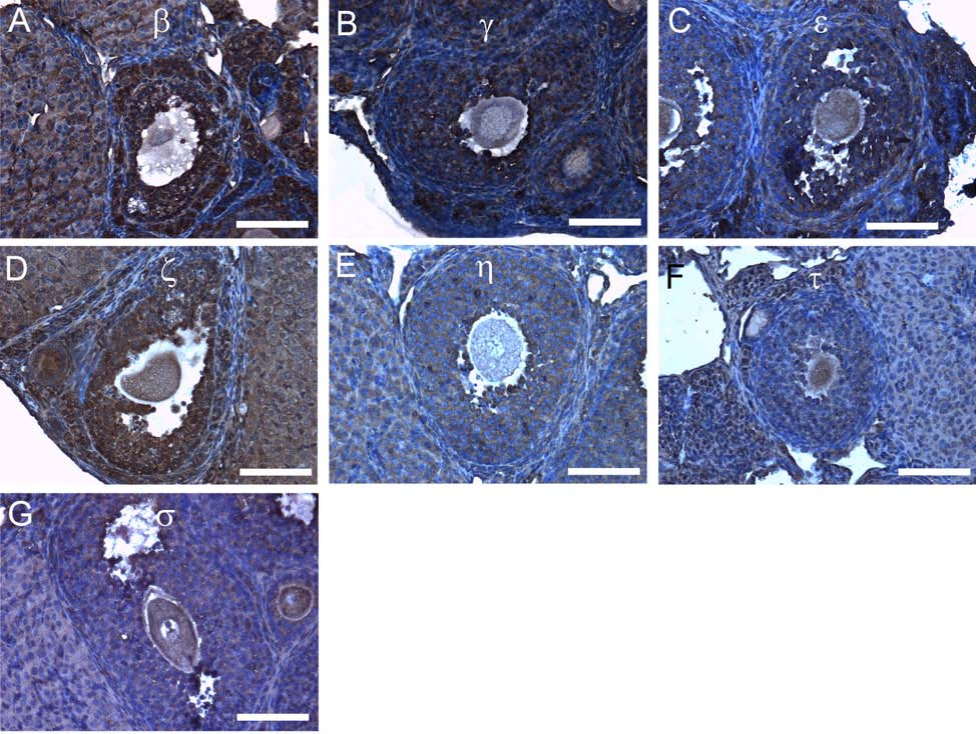
**Representative immunohistochemistry images of 14-3-3 protein isoforms in atretic follicles of adult mouse ovaries**. (A) 14-3-3 β. (B) 14-3-3 γ. (C) 14-3-3 ε. (D) 14-3-3 ζ. (E) 14-3-3η. (F) 14-3-3 τ. (G) 14-3-3 σ. All scale bars indicate 100 μm.

We show here by immunohistochemistry that all seven isoforms of 14-3-3 protein were detected in cells of the ovary. Follicles at various stages of development exhibit some common features of expression of the isoforms. All isoforms of 14-3-3 were detected, to varying extents, in the oocyte and cumulus cells surrounding the oocyte, mural granulosa cells, theca interna and theca externa of all follicular stages examined, as well as in cells of the *corpus luteum *(Figures 4, 5, 6, 7, 8, 9 and 10). In each of the follicular stages studied, all the isoforms appear to be expressed in the cytoplasm of the oocytes and to some extent in the corresponding germinal vesicles (Figures 4, 5, 6, 7, 8,9 and 10). For all isoforms, staining appeared more intense in the cytoplasm than in the nuclei of somatic cells in granulosa and theca layers as well as in cells of *corpora lutea *(Figures 4, 5, 6, 7, 8,9 and 10). Cells within *corpora lutea *were also found to have relatively higher amounts of expression of all of the isoforms as compared to surrounding interstitial cells F in (Figures 4, 5, 6, 7, 8,9 and 10). The isoform 14-3-3τ appears to be expressed at lower levels in somatic cells when compared to oocytes (Figure 9A-E). Atretic follicles, characterized by intensely stained pyknotic (apoptotic) and/or lytic cells, exhibit prominent accumulation of all isoforms of 14-3-3 (Figure 11A-G).

While the specific activities of 14-3-3 proteins in ovarian function are largely unknown, these proteins could play a significant role. For example, 14-3-3τ, shown to be present in human granulosa cells, has been shown to bind to the human follitropin (follicle stimulating hormone, FSH) receptor suggesting a role for 14-3-3 proteins in follitropin signaling in granulosa cells [24,25]. The reason for intense 14-3-3 staining in apoptotic cells is not known. 14-3-3 is generally associated with anti-apoptotic functions [26]. 14-3-3 proteins appear to be involved in several ways in promoting cell survival, for example 14-3-3 has been found to enhance the activity of proteins with proliferative or survival roles, such as members of the Raf family, while antagonizing the activity of proteins that promote cell death [3,27]. There is some suggestion that 14-3-3σ is associated with the survival of granulosa cells and inhibition of 14-3-3 interaction with target proteins, without regard to isoform, promotes apoptosis in these cells [28,29].

**Figure 12:**
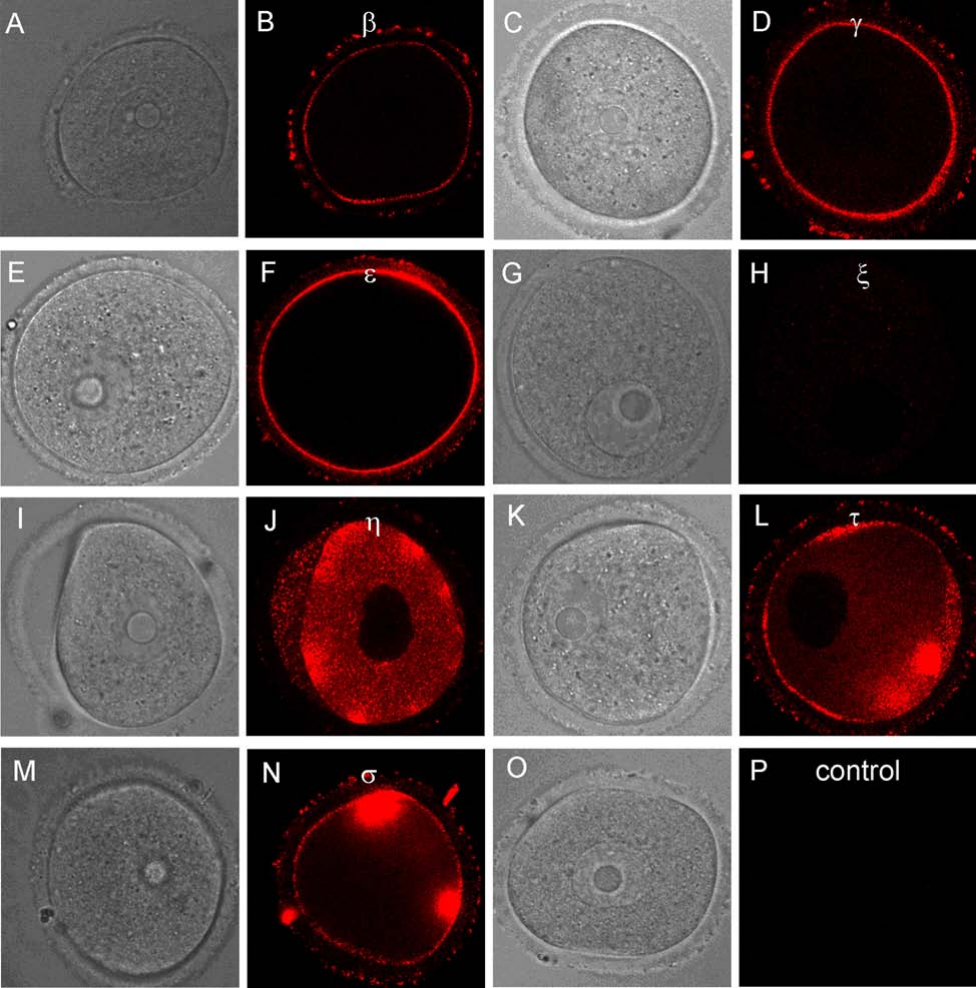
**Representative immunocytochemistry images of 14-3-3 protein isoforms along and in the *zonae pellucidae *of cumulus-free oocytes isolated from ovaries of adult mice**. The zona-intact cells were fixed in paraformaldehyde but not treated with detergent (see Methods). Paired images of an oocyte (left image is brightfield and right is immunofluorescence) indicate staining along the zona and/or the cell membrane for all isoforms except 14-3-3ζ. Note: the intent of this part of the study was not to examine the intracellular distribution of the isoforms (see Figure 3 for those experiments) since the cells were not permeabilized to permit complete antibody penetration; however, cells may be partially permeabilized by fixation accounting for the detection of intracellular proteins in some cells. (A,B) 14-3-3 β. (C,D) 14-3-3 γ. (E,F) 14-3-3 ε. (G,H) 14-3-3 ζ. (I,J) 14-3-3 η. (K,L) 14-3-3 τ. (M,N) 14-3-3σ. (O,P) Control image without primary antibody addition.

Several characteristic differences in expression of 14-3-3 protein isoforms in different stages of follicular development were observed by immunohistochemical staining. Detection of 14-3-3β, 14-3-3γ, 14-3-3ε, 14-3-3τ and 14-3-3σ in *zonae pellucidae *of oocytes by immunohistochemical staining indicates secretion of some 14-3-3 proteins into the *zonae *and perivitelline space (Table 1; C in Figures 4, 6, 9 and 10). To examine this by another method, we isolated zona-intact oocytes and viewed them by immunofluorescence. Such immunocytochemical staining detected the same isoforms along the membranes of the isolated oocytes and within or along the *zonae pellucidae *of oocytes (Figure 112B, D, F, L and N), indicating secretion of those 14-3-3 isoforms by the oocyte or associated follicle cells. Control oocytes processed for fluorescence microscopy without the addition of primary antibody showed no fluorescence above background (Figure 12O-P).

**Table 1:** Characteristic differences in expression of 14-3-3 protein isoforms in the different stages of follicular development as observed by immunohistochemical staining of adult mouse ovarian sections

Feature	Isoform	Stage of Ovarian Follicle		
		**Secondary**	**Early Antral**	**Graafian**

**Expression** **in* zonae pellucidae*** **around** **oocytes**	14-3-3 β 14-3-3 γ 14-3-3 ε 14-3-3 ζ 14-3-3 η 14-3-3 τ 14-3-3 σ	++ + ++ + - ++ ++	++ + ++ + - ++ ++	++ + ++ - - ++ ++

**Prominent** **lack of expression** **in peripheral mural granulosa** **cells**	14-3-3 β 14-3-3 γ 14-3-3 ε 14-3-3 ζ 14-3-3 η 14-3-3 τ 14-3-3 σ	Yes Yes Yes No Yes Yes Yes	Yes No No No No No No	Yes Yes Yes No No Yes Yes

**Marked accumulation** **in granulosa cells surrounding the antrum**	14-3-3 β 14-3-3 γ 14-3-3 ε 14-3-3 ζ 14-3-3 η 14-3-3 τ 14-3-3 σ		Yes Yes No No No No Yes	Yes Yes Yes Yes Yes Yes Yes

'++' = Considerable expression; '+' = Some expression;

'-' = Non-detectable expression; 'Yes' = Presence of the feature; 'No' = Feature not detectable

The function, if any, of extracellular 14-3-3 surrounding the oocyte is not yet known. We do not believe this is an artifact of fixation since the appearance of the extracellular 14-3-3 was found by two different methods and the absence of staining for some isoforms argues against nonspecific artifacts. We did not find extracellular 14-3-3 proteins associated with mature, ovulated eggs, which also suggests that this is not an artifact (data not shown). While the 14-3-3 proteins are generally known as intracellular proteins, some 14-3-3 proteins are known to be secreted by somatic cells; for example, it has been known for some time that 14-3-3 proteins are found in the cerebrospinal fluid [30] where they may serve as markers for Creutzfeldt-Jakob disease. Keratinocytes secrete 14-3-3σ which induces expression of matrix metalloproteinase 1 (MMP1 or collagenase) in fibroblasts [31]. Three isoforms, 14-3-3 σ, 14-3-3 γ and 14-3-3ζ, are secreted by corneal epithelial cells and ocular cell lines [32]. The14-3-3ζ protein is secreted by certain tumor associated inflammatory cells [33].

Another notable difference in the distribution of 14-3-3 isoforms in ovarian folliculogenesis is prominent lack of expression 14-3-3β, 14-3-3γ, 14-3-3ε, 14-3-3τ, 14-3-3η and 14-3-3σ in the peripheral mural granulosa cells, as compared to other cells of secondary, early antral and/or Graafian follicles (Table 1; C in Figures 4, 5 and 6 and 8, 9 and 10). At early antral stages, intense staining was noted for 14-3-3β, 14-3-3γ and 14-3-3σ in granulosa cells surrounding the antrum (Table 1; D in Figures 1, 2 and 10). At the Graafian stage, all isoforms appear to accumulate in these cells (Table 1; E in Figures 4, 5, 6, 7, 8, 9 and 10).

## Conclusions

Seven isoforms of the protein 14-3-3 are expressed in the female mouse germ cells and in cells of mouse ovarian follicles at various stages of development. There are characteristic differences in the relative amount and distribution of 14-3-3 proteins in oocytes and eggs. Protein 14-3-3η, for example localizes, in part, to the meiotic spindle in eggs. A number of isoforms appear to be extracellular and associated with the *zona pellucida *in ovarian oocytes. The distribution of some 14-3-3 isoforms within cells of the ovary differs, for example with peripheral mural granulosa cells expressing some isoforms and not others. All 14-3-3 isoforms appear to be present in relatively greater amounts in cells lining the antral cavity of Graafian follicles and in granulosa cells of atretic follicles. These results will enable further research investigating 14-3-3 interactions with other key proteins involved in ovarian development and gamete function.

## Methods

### Collection of Oocytes and Eggs and Preparation of Ovarian Tissue Extracts

All mice used in the present experiments were housed and used at Kent State University under an approved Institutional Animal Care and Use Committee protocol following the National Research Council's publication Guide for the Care and Use of Laboratory Animals. Oocytes and eggs were collected as previously described [12]. CD1 mice (2.5 months old) were injected with 7.5 IU eCG and, 44-48 h later, the ovaries were removed and repeatedly punctured with a 26-gauge needle to rupture follicles. Cumulus cell-enclosed oocytes were isolated and the cumulus cells were removed by repeated pipetting though a small-bore pipette. Fully-grown oocytes with intact nuclei (germinal vesicles) were cultured in MEM containing 0.1 mg/ml dibutyryl cAMP to prevent spontaneous oocyte maturation. Mature, metaphase II-arrested eggs were obtained from mice 13-14 h following superovulation by injection of 7.5 IU hCG which was preceded by a priming injection of 7.5 IU eCG injection 48 h earlier. The cumulus cells were removed with 0.3 mg/ml hyaluronidase. *Zonae pellucidae *from oocytes and eggs thus collected were removed by a brief treatment in acid Tyrode's solution (0.14 M NaCl, 3 mM KCl, 1.6 mM CaCl_2_.2H_2_O, 0.5 mM MgCl_2_.6H_2_O, 5.5 mM glucose, and 0.1% PVA, pH 2.5). Cells were rinsed in MEM and prepared for use in Western blot or immunocytochemistry as described below.

### SDS-PAGE, Western Blotting and Protein Analysis

14-3-3 isoforms were identified through multiple Western blots (using standard Western blotting procedures), each testing for one of the seven isoforms in protein extracts. Oocytes and eggs were rinsed in MEM, counted, and transferred to Tris-buffered saline (TBS; 25 mM Tris-HCl [pH 7.5] and 150 mM NaCl) containing 0.1% PVA. Cells were removed from the TBS, lysing buffer was added, and the cell lysates were quick-frozen in ethanol/dry ice and stored at -70°C until use. The lysis buffer contained 10 mM Tris-HCl [pH 7.2], 1 mM EDTA, 1 mM EGTA, 0.1% (v/v) b-mercaptoethanol, 1% (v/v) Triton X-100, protease inhibitors (1 mM PMSF, 0.1 mM TPCK, 10 μM leupeptin, 1 μM pepstatin A, and 75 nM aprotinin), and phosphatase inhibitors (1 mM Na_3_VO_4_, 100 nM calyculin A, 10 mM beta-glycerophosphate, and 5 mM sodium pyrophosphate).

Proteins from the lysates of 200 oocytes or 200 eggs were separated by SDS-PAGE using a 4% stacking, 12% resolving polyacrylamide gel and electrophoretically transferred to Immobilon-P PVDF membrane (Millipore Corp.). The membranes were incubated in blocking buffer (5% milk in TBS, 0.1% Tween-20) and then with primary antibodies overnight at 4°C, washed and incubated with secondary antibody and imaged by chemiluminescence with an enhanced chemiluminescence kit according to the manufacturer's instructions (GE Healthcare Life Sciences) using the Fujifilm LAS-3000 luminescent image analyzer. Protein extracts of ovaries from adult mice were included for comparison, loading identical amounts of protein extract for all isoform immunoblots. Brain extract, loaded with the same amount of protein extract for each isoform, was used as a positive control for all isoforms of 14-3-3 except 14-3-3σ for which skin extract was used as positive control. This is because all isoforms of 14-3-3 have been indentified in brain except 14-3-3η which has been detected in skin [15]. Ovaries from unprimed adult mice (2.5 months old), brain and skin tissues were homogenized in a buffer containing (10 mM Tris pH 7.0, 1 mM EDTA, 1 mM EGTA, 0.16% benzamidine hydrochloride, 14 mM beta-mercaptoethanol, 1 mM PMSF and 0.1 mM TPCK) pH 7.2, using a mechanical homogenizer. The homogenized cell lysates were then centrifuged at 16,000 μg for 30 min and the supernatants containing the total soluble protein extracts were used for SDS-PAGE and Western blotting.

We used the commercial rabbit anti-14-3-3 isoform panel (PAN017) from AbD Serotec, for Western blotting as well as immunocytochemical and immunohistochemical staining (see below) of the different 14-3-3 isoforms in mouse ovaries, oocytes and eggs. The immunogens, against which the antibodies were raised, were synthetic peptides corresponding to acetylated N-terminal sequences of sheep 14-3-3 proteins. They are raised against synthetic peptides corresponding to the following acetylated N-terminal sequences of sheep 14-3-3 isoforms: 14-3-3β, TMDKSELVC; 14-3-3γ, VDREQLVQKAC; 14-3-3ε, MDDREDLVYQAKC; 14-3-3ζ: MDKNELVQKAC; 14-3-3η, GDREQLLQRARC; 14-3-3 τ, MEKTELIQKAC; 14-3-3σ, MERASLIQKAC.

Two additional antibodies to 14-3-3β and 14-3-3ζ were also used to confirm the presence of these isoforms (rabbit anti-14-3-3β, sc-628 and rabbit anti-14-3-3 ζ sc-1019, Santa Cruz Biotechnology). All antibodies were used at a dilution of 1:1,000 in blocking buffer (5% milk in 1X TBS, 0.1% Tween-20). The secondary antibody for all experiments was HRP-conjugated goat anti-rabbit (Genscript; 1 mg/ml stock) used at a dilution of 1:2,000 in the blocking buffer.

The relative abundance of each 14-3-3 isoform in oocytes and eggs was determined in a semi-quantitative manner. Proteins from 200 oocytes and 200 eggs were separated by electrophoresis on the same gel, transferred to membrane at the same time and immunoblotted together under the same conditions using one of the six 14-3-3 antibodies. This procedure was repeated two additional times with the same antibody. There will be inherent differences in the intensities of bands for each immunoblot processed, but for a given blot the intensity of the egg band can be compared to the intensity of the oocyte band. To summarize the three experiments for each of the six isoforms, the band intensities for oocyte and egg lanes in a given blot were analyzed using NIH image and compared to each other. The intensity of the oocyte band was normalized to 100% for each blot and the egg intensity (reflecting the relative protein amount) was then expressed as a percentage of the oocyte intensity for each of the three blots analyzed for each isoform. Note that no comparison should be drawn for the band intensities among the isoforms since the antibodies detecting the isoforms were different from each other, with possible differences in their affinities.

### Immunocytochemistry of oocytes and eggs

Oocytes and eggs were fixed in freshly prepared 3.7% paraformaldehyde for 30-60 min, washed in PBS-PVA (PBS containing 1% PVA), permeabilized with 1% Triton X-100 to promote antibody penetration, washed in PBS-PVA, then treated with blocking buffer (5% normal goat serum in PBS-PVA), and incubated overnight with each of the primary antibodies for 14-3-3 isoforms (rabbit anti-14-3-3 isoform panel PAN017, AbD Serotec; diluted 1:200 in 1% goat serum blocking buffer). Following washing, the cells were incubated with Cy3-conjugated goat anti-rabbit secondary antibody (Jackson ImmunoResearch Laboratories) diluted 1:200 in blocking buffer for several hours, washed again and transferred to an anti-fade solution (SlowFade; Invitrogen). All cells were imaged with the Olympus Fluoview FV500 confocal microscopy system using a 60× oil immersion lens and various confocal zooms; the scale bars on the images indicate the final magnification. Images were captured and examined at multiple confocal planes. The representative images shown here are primarily images at the plane of the optical equator. For each isoform experiment, 5-7 oocytes and 5-7 eggs were examined at the same time under the same staining and imaging conditions. The experiment was repeated twice for each isoform.

In oocytes, the nucleus is readily discernible in fluorescence images or the corresponding brightfield images. In eggs, the location of the condensed metaphase II chromosomes can sometimes be determined by a bulge at one pole of the egg or by the absence of fluorescence causing an outline of the unlabeled chromosomes. In an additional experiment using the 14-3-3η antibody, condensed meiotic chromosomes in eggs were identified by staining with the DNA-staining Hoechst dye (0.001 mg/ml) and imaged with conventional epifluorescence microscopy (Figure 3N, inset). In several experiments, eggs were simultaneously incubated in both the 14-3-3η isoform antibody and an antibody to alpha-tubulin to identify the meiotic spindle microtubules (rat anti-alpha tubulin; sc-69970; 200 μg/ml diluted 1:200 in 1% blocking buffer; Santa Cruz Biotechnology). The secondary antibody was FITC-conjugated goat anti-rat from (Jackson Laboratories) (diluted 1:200 in blocking buffer). In all cases, control oocytes and eggs were incubated in secondary antibody alone and imaged using the same confocal settings as used for experimental, antibody-labeled cells. Background fluorescence was minimal.

An additional set of immunofluorescence experiments was performed to examine the presence of extracellular 14-3-3 isoforms associated with isolated oocytes. The immunofluorescence method was used as described above, but with several changes. In this case, the *zonae *were not removed before fixation in 3.7% paraformaldehyde and the cells were not treated Triton X-100 to permeabilize the cell membranes following fixation. All other staining procedures were the same. We wanted to look at the possibility of secreted proteins in oocytes. This experiment was not intended to examine the intracellular distribution of isoforms (see above methods and Figure 3 for that experiment); however, we have known for many years that mouse oocytes and eggs sometimes are partially permeabilized on fixation alone (no detergent added). Therefore some staining within the oocytes may occur, but is not be a complete representation since the permeability of cells may vary from cell to cell and is not uniform within a cell.

### Immunohistochemistry of tissue sections

Ovaries, brain and skin tissues were collected from unprimed adult mice (2.5 months old) and fixed in 4% paraformaldehyde in PBS overnight. They were then dehydrated through a graded series of ethanol followed by two changes of CitriSolv and embedded in paraffin. Multiple microtome tissue sections of 6 μm thickness were transferred to slides pre-coated with poly-L-lysine. Following removal of paraffin and rehydration, antigens were recovered by boiling the sections for 1 min, three times in Antigen Retrieval Citra solution (#HK086-5K, Biogenex) with intermittent cooling. Tissue sections were then cooled at room temperature for 30 min and then washed in deionised water for 5 min. Endogenous peroxidase was blocked by incubation of tissue sections in 0.3% hydrogen peroxide for 30 min. Tissues were then incubated for 20 min in PBS blocking buffer or 0.15% normal goat serum (VectaStain Elite ABC Kit, #PK-6101, Vector Laboratories). Tissues sections were incubated overnight at 4°C in a humidified chamber with each of the different primary antibodies (14-3-3 isoform panel PAN017, AbD Serotec, see above) diluted in blocking buffer at dilutions recommended by the manufacturer and a prior study [34]:14-3-3β 1:600, 14-3-3γ 1:800, 14-3-3ε 1:400, 14-3-3ζ 1:400, 14-3-3η 1:3,200, 14-3-3τ 1:60, and 14-3-3σ 1:100. The same dilutions were used for positive control sections. The rabbit serum used in matching negative control sections, were immunostained at a dilution identical to that used in corresponding sample sections for the isoforms.

Following antibody incubation, slides containing tissue sections were washed in buffer (PBS, pH 7.5) for 5 min, incubated for 30 min in biotinylated secondary antibody (VectaStain Elite ABC Kit, Vector Laboratories) diluted in blocking buffer and washed again in buffer for 5 min. This was followed by incubation of the tissues for 30 min in VectaStain Elite ABC Reagent (prepared according to the manufacturer's instructions; Vector Laboratories). DAB substrate was prepared using Vector Laboratories DAB peroxidase substrate Kit (SK-4100) according to instructions specified in kit. Tissues were treated with DAB substrate for 3-4 min until development of optimum brown color, rinsed with tap water and counter-stained with Hematoxylin.

**Figure 13:**
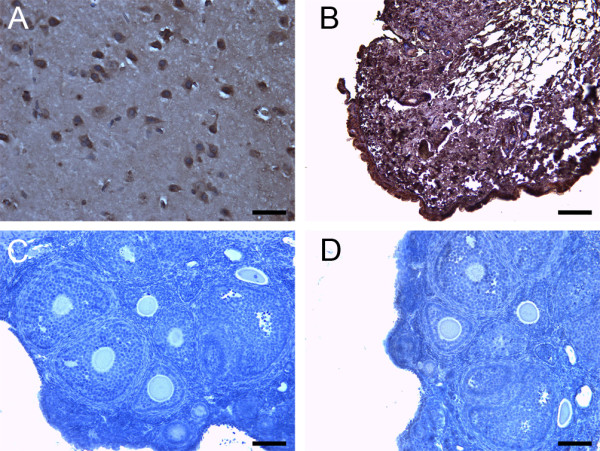
**Representative control immunohistochemistry images for the different 14-3-3 protein isoforms in tissue sections**. (A) Example of a positive control brain section obtained for all 14-3-3 isoforms except for 14-3-3 σ. (B) Skin positive control for 14-3-3 σ. (C) Example of a negative control section with no primary antibody obtained for all isoforms. (D) Example of a negative control with rabbit serum in place of primary antibody obtained for all isoforms. All scale bars indicate 100 μm.

The ABC-immunostaining method produces minimal background staining. To examine the background staining, two sets of negative controls were used. One set of tissue sections was processed without the addition of primary antibodies. Since the primary antibodies were rabbit polyconlonals, the other control set utilized tissue sections incubated in normal rabbit serum (Jackson ImmunoResearch) prior to incubation with the secondary antibodies. In both cases, control sections did not show brown staining, confirming specific localization by this method. Brain tissue sections were used as positive control for all isoforms of 14-3-3 except 14-3-3σ for which skin tissue was used as positive control (see Figure 13). This is because all isoforms of 14-3-3 have been identified in brain with the exception of 14-3-3σ [13]. Skin sections were used as positive control for 14-3-3σ since 14-3-3σ is known to be found in epidermis [15] (see Figure 13). For each isoform, dilutions of primary antibodies or rabbit serum for positive and negative controls were kept identical. Each 14-3-3 isoform was studied individually in a set of 3 separate stained ovarian tissue sections, along with simultaneous staining of appropriate positive and negative controls. In addition the studies were repeated on sections obtained from the ovary of a different unprimed adult mouse of the same age. The characteristic features for all 14-3-3 isoforms, as identified by immunohistochemical staining, were examined and tabulated (Table 1) independently by three individuals with agreement on the observations.

## Abbreviations

ABC: Avidin-biotin complex; CDC25B: Cell division cycle 25 homolog B; Cy3: Cyanin3; DAB: 3,3' Diaminobenzidine; eCG: Equine chorionic gonadotropin; FITC: Fluorescein isothiocyanate; hCG: Human chorionic gonadotropin; HRP: Horse radish peroxidase; MEM: Minimal essential medium; PADI6: Peptidyl arginine deiminase 6; PMSF: Phenylmethylsulfonyl fluoride; PVDF: Polyvinylidene fluoride; TBS: Tris buffered saline; TPCK: Tosyl phenylalanyl chloromethyl ketone; Tris-HCl: Tris-hydrochloride; YWHA: Tyrosine 3-monooxygenase/tryptophan 5-monooxygenase activation protein.

## Competing interests

The authors declare that they have no competing interests.

## Authors' contributions

SD, JLM and DK conducted all practical work. All authors reviewed the data and combined to draft the manuscript. All authors read and approved the final version of the manuscript.
